# Knowledge, Awareness and Practices Regarding Cystic Echinococcosis among Livestock Farmers in Basrah Province, Iraq

**DOI:** 10.3390/vetsci5010017

**Published:** 2018-02-06

**Authors:** Mohanad F. Abdulhameed, Ihab Habib, Suzan A. Al-Azizz, Ian Robertson

**Affiliations:** 1 Veterinary Public Health and Epidemiology Section, School of Veterinary and Life Sciences, College of Veterinary Medicine, Murdoch University, Perth 6150, Australia; m.abdulhameed@murdoch.edu.au (M.F.A); i.robertson@murdoch.edu.au (I.R); 2 Department of Parasitology, College of Veterinary Medicine, University of Basrah, Basrah 61004, Iraq; suzanalazizz@yahoo.com; 3 High Institute of Public Health, Alexandria University, Alexandria 21516, Egypt; 4 China-Australia Joint Research and Training Center for Veterinary Epidemiology, Huazhong Agricultural University, Wuhan 430072, China

**Keywords:** cystic echinococcosis, Iraq, questionnaire survey, knowledge, livestock

## Abstract

Cystic echinococcosis (CE) is an endemic neglected parasitic zoonosis in many of the countries of the Middle East. The disease poses a remarkable economic burden for both animals and humans. In this study, we conducted a questionnaire survey among livestock farmers in Basrah province, southern Iraq, in order to evaluate their knowledge and awareness about CE, and to understand some of the risky practices that could contribute to spread and persistence of such disease. Of the interviewed participants (N = 314), 27.4% owned dogs on their farms. Among farmers owning dogs, 76.7% (66/86) never tied up their dogs, and 43% (37/86) indicated feeding uncooked animal viscera to their dogs. The majority (96.5%) of the farmers indicated that they did not de-worm their dogs at all. Only 9.8% (31/314) of the respondents indicated eating raw leafy vegetables without washing. Added to that, 32% of the interviewees indicated that they source water for domestic use from a river; meanwhile 94.3% (296/314) of them do not boil water before using it for domestic purposes. Half of the interviewed livestock farmers in Basrah were not aware about how humans get infected with CE disease, and 41.4% (130/314) did not even realize that CE is a dangerous disease to human health. Almost one in three of the respondents who owned dogs on their farms viewed de-worming of their dogs as a low priority practice. This study highlights the gap in knowledge and awareness about CE among the study population. Risky practices associated with dog keeping management and food and water handling practices were identified. The insight from this research could be used to improve the delivery of a health education message relevant to cystic echinococcosis control at the human-animal interface in Iraq.

## 1. Introduction

Cystic echinococcosis (CE) is a neglected zoonotic disease caused by the tapeworm *Echinococcus granulosus*. The disease is reported worldwide [1,2]. In endemic regions, CE poses a remarkable One Health challenge due to the economic losses in animals combined with the high risk of morbidity in humans [3]. At a global level, it has been estimated that there are more than one million human CE cases with a disease burden between 1 and 3.6 million disability-adjusted life years (DALYS) [4].

CE is particularly highly endemic in most of the countries of the Mediterranean basin, including North Africa and the Middle East. In Libya, the incidence rate of human CE was estimated at 4.2 cases per 100,000 inhabitants [5]. In Iraq, the incidence has been reported at 6.3 per 100,000 inhabitants [6]. In Egypt, the incidence rate of human CE varied between 1.3 and 2.6 cases per 100,000 inhabitants [7]. An incidence rate has been reported in Tunisia to be as high as 12.6 per 100,000 inhabitants [8]. Human infection in endemic regions is influenced by different biotic and abiotic factors, and also depends on a number of behavioural and socio-economic variables [2,9].

According to a review by Sadjjadi [10], CE is considered as hyper endemic in humans in Iraq. Several human cases with CE have been reported across Iraq [11,12,13]. However, there is no national surveillance data regarding the actual number of CE cases in Iraq. In Basrah, southern Iraq, the incidence rate of human CE has been estimated as 5.6 cases per 100,000 inhabitants [14,15]. The prevalence of *Echinococcus granulosus* in stray dogs in Basrah was recorded at 14.7%, while the prevalence of hydatid cysts in slaughtered sheep in Basrah was reported as 22% [16].

Transmission of CE is influenced by socio-economic and cultural conditions of a community. For instance, home slaughtering practice, improper disposal of internal organs of livestock to dogs, neglected de-worming of dogs, feeding dogs with condemned offal are all considered among the common practices associated with increasing prevalence and risk of exposure of domestic animals to CE [17,18,19]. Added to that, the low level of education is also speculated to be a risk factor in human CE [20]. Dogs are the major source of infection to humans, and the majority of documented human CE cases are caused by *E. granulosus* with a life cycle that occurs mainly within a rural setting between sheep and shepherd dogs [21].

To achieve an effective control program of CE, it is important to evaluate the level of knowledge about the disease, awareness regarding the preventive measures, and risky practices that spread the disease within the community. There is no published research investigating these topics in Iraq. For these reasons, we conducted a survey to investigate the disease-related knowledge, awareness and practices among livestock farmers in Basrah, southern Iraq. The insight from this research could be used to develop health promotion tools and to improve the delivery of an efficient health education message relevant to CE control at the human–animal interface in Iraq.

## 2. Materials and Methods

### 2.1. Study Area

The study subjects were livestock farmers in Basrah province. Basrah is the third largest province in Iraq and located in the south of the country and borders Iran, Kuwait, and Saudi-Arabia. Basrah is in a fertile agricultural region, with major agriculture and livestock production. The province had an estimated human population of 2,403,301 million, in which the rural-urban proportion is distributed at 20.1–79.9%, respectively [22].

### 2.2. Study Design

Throughout this cross-sectional study, 320 farmers were enrolled from 20 villages distributed over the six counties of Basrah (Abu Al-Kasib, Al-Midaina, Al-Qurnah, Al-Zubair, Shat Al-Arab, and Al-Basrah). The sample size was based on 95% confidence limits at the precision of 5% and assuming response distribution of 30%. It was not possible to execute a random sampling approach from the study subjects, given the political and tribal conflicts in Iraq during the field work period (March–July 2016). Hence, we adopted in this study a convenience targeted sampling approach. After coordination with local veterinary authorities, the inclusion criterion for the targeted sampling was the possibility of gaining a secured access to the targeted villages, based on security situation assessment at the time of the study. Local veterinarians and tribal leaders in each village assisted in introducing the research team to the farmers’ community and helped in explaining the purpose of this survey. In each village, farmers were chosen using a chain-referral sampling method in which the first select farmer provided information about the next available farmer in the area until the required number of respondents had been achieved. Out of 320 farmers, those were approached by the research team; only six farmers were not willing to participate, hence in total 314 farmers accepted to fill in this questionnaire data collection tool during a face-to-face interview visits to their farm.

### 2.3. Questionnaire Design and Administration

The study instrument was a questionnaire which comprised of three sections with approximately 30 questions. Part 1 was related to socio-demographic characteristics; part 2 was related to practices towards CE prevention; and part 3 concerned knowledge and awareness about CE infection and transmission sources. The questions were either closed-ended or dichotomous, and the questionnaire was pre-tested by the authors to allow for improvements. Prior to the interview, a verbal consent form was obtained from each participant. The questionnaire survey was administrated in the local language (Arabic). A copy of the questionnaire could be obtained from the corresponding author. Data were coded and stored in Microsoft Excel 2000 (Microsoft, Redmond, WA, USA) sheets, and descriptive data analysis was performed using STATA software v.14 (StataCorp LLC, College Station, TX, USA).

### 2.4. Ethics Statement

This study was approved by the Human Ethics Review Committee of Murdoch University, Perth, Australia (Permission number: 034/2016). Official written approvals from the Ministry of Health in Iraq and from Basrah Health Directorate were obtained before commencement of the field work.

## 3. Results

### 3.1. Socio-Demographic Characteristics of the Study Population

Age of the interviewed farmers ranged from 18 to 84 years (mean: 45 ± 2 standard deviation), and the vast majority (99.1%) were males (Table 1). Regarding the farmers’ level of education, 50% of the farmers had completed primary school, and 25% of them were unable to read and write. Working as a farmer was a secondary occupation in almost 20% of the interviewees (Table 1). Cattle, alone or integrated with sheep and buffalo, was the main livestock species owned by the farmers interviewed in this study (Table 1).

### 3.2. Practices towards CE Prevention

Of all the interviewed farmers, 27.4% (86/314) indicated owning dogs on their farms (Table 2). Our result highlights some negligent dog management practices. For instance, among farmers owning dogs, 76.7% (66/86) never tied up their dogs. Added to that, 43% (37/86) of the interviewed farmers indicated that they feed uncooked animal viscera to their farm dogs (Table 2). The majority of the farmers (95.3%) answered that they tend to leave dog fecal droppings unattended on the ground of the farm wherever they are. The interviews also revealed that the vast majority of the farmers (96.5%) do not de-worm their dogs.

More than half (60%) of the farmers indicated that their dogs frequently come in close contact with the livestock raised on their farms. The questionnaire results also highlighted some unsanitary food and water handling practices among the interviewed farmers (Table 2). It is worth highlighting that among all the respondents, 9.8% (31/314) indicated eating raw leafy vegetables just after peeling the outer leaves but without considering any kind of washing with water. Almost a third (32%) of the livestock farmers indicated that they source water for domestic purposes from a nearby river, and 94.3% (296/314) answered that they do not boil water sourced from the river (Table 2).

### 3.3. Knowledge and Awareness about CE Infection and Transmission Sources

The interviews revealed that 40.8% (128/314) of the study subjects did not know about the possibility of transmission of certain diseases (zoonoses) between livestock and humans. However, 70.7% (222/314) of the farmers answered that they had previously heard about CE disease; despite that, 41.4 (130/314) of them did not know that CE can cause harm to human health (Table 3). Almost half of the interviewed livestock farmers in Basrah were not aware on how humans acquire the infection with CE. Added to that, 64.4% (202/314) of them were not aware that livestock animals could also get infected and act as carrier for the parasite causing CE. Almost one third of the farmers who owned dogs answered that they regard de-worming of their dogs as a low priority practice to do and failed to appreciate the importance of de-worming for either dogs or human health (Table 3).

## 4. Discussion

In this study, despite several obstacles due to political and security instability in Basrah province, we were able to administrate face-to-face questionnaires with 314 livestock farmers. To the best of the authors' knowledge, this is the first study in Iraq to collect data on livestock farmers’ knowledge about CE, awareness regarding the preventive measures against such important neglected zoonosis, and risky practices that could contribute to spread and persistence the disease. The population interviewed in this study (livestock farmers) is an important target for any One Health education campaign aiming at raising awareness on CE at the human–animal interface. Sound understanding of the epidemiology of CE in livestock-raising communities is a key factor in limiting the transmission cycle of this important neglected zoonosis to humans [2,23].

Several potential risky practices have been underlined among the livestock farmers community interviewed in this study, notably practices related to dog management on farms. Almost three-quarters of the farmers never tie up their dogs. This finding reflects a poor awareness among the farmers in Basrah regarding the role of dogs in CE transmission. A study from Libya demonstrated that untying dogs was a significant risk factor for increasing the positivity of detecting *Echinococcus granulosus* in dogs’ faecal samples [24]. In addition to not tying up their dogs, the majority of the interviewees did not de-worm their dogs, and half of them indicated feeding uncooked viscera to their dogs. Such risky practices have been among the most important factors that increase contamination of the environment with faeces containing *Echinococcus* eggs [23]. Similar findings have been documented in other CE endemic settings. A study in Sardinia (Italy) reported that the majority of the interviewed farmers used raw offal, after home slaughtering, for feeding their dogs [25]. In addition, a study in Tibet demonstrated that feeding dogs with uncooked viscera is a risk factor for increasing the likelihood of human infection with *E. granulosus* [26].

Our study also revealed that the majority of the interviewed farmers tend to leave dogs faeces unattended on the farm ground. Such unhygienic practice might carry potential risk mainly for children who tend to play and crawl on the ground. Added to that, this could also increase the chance of dog faeces contamination of other crops, accidentally or as fertilizer. It has been reported that *Echinococcus* eggs that deposited in the soil could stay viable for up to a year [27]. On another aspect, more than half of the dogs’ owners revealed that their dogs have frequent contact with the livestock animals on their farms. Similar to our finding, a study in Portugal indicated that dogs have close contact with livestock among approximately 60% of the interviewed farmers [28]. Positive CE copro-antigen results were mainly reported in working dogs such as hunting, guard or shepherd dogs that presumably are more likely to roam freely [29,30].

In highly endemic areas it is quite possible for individuals to contract CE through indirect transmission through contaminated food or water [31,32]. Our results show that approximately 10% of the respondents indicated eating raw leafy vegetables without washing. Although not a consistent finding, there are studies that indicate an association between CE human infection and eating homegrown vegetables presumably have been contaminated by dog faeces [33]. One third of the farmers interviewed in our study reported using water supplied from a nearby river. Unsafe water supply has been also found to be associated with infection with CE, and this may be due to water contamination with dog faeces [31,34]. Adequate hygienic handling practices and heat treatment (cooking food or boiling water) should contribute to minimizing the risk of foodborne echinococcosis.

The results of this study indicate that CE seems to be not a familiar disease to the livestock farmers’ community in Basrah, southern Iraq. Around 40% of the farmers did not know how CE disease could be transmitted to the human, and even did not realize that CE is dangerous to human health. In Jordan, a neighboring country to Iraq, awareness regarding CE in rural communities was higher (86%) compared to what we experienced in the present study in Basrah [35]. In our study, half of the interviewed farmers were not aware of how a human can acquire the disease and more than 60% of the farmers were not aware that livestock could become infected with CE. Compared to our finding, a study in Morocco concluded an even lower level of awareness regarding CE, with only 20% of the interviewees realizing that dogs play a role in the transmission of CE in humans and animals [36]. Collectively, these findings call for an urgent need to strengthen the health education strategy among rural communities and livestock farmers in Iraq. It was quite worrisome to reveal that most of the livestock farmers in this study had a negative attitude toward de-worming their dogs, given that the majority did not realize the benefits of dog de-worming for both animal and human health. We hypothesize that the cost and availability of anthelmintic is probably one of the main reasons of its quite low frequency of use among farmers in Basrah.

## 5. Conclusions

After decades of war, sanctions and political instability, Iraq has faced socio-economical-political challenges. The level of education and communication among rural communities and healthcare providers has dropped largely, which has influence on many education and health indicators in the study area. This study highlights the gap in knowledge about cystic echinococcosis among the livestock farmers community in Basrah. The awareness regarding specific CE preventive measures was not optimal. Risky practices concerning dog keeping management on farms and on food and water handling practices were identified. In Basrah, the incidence rate of CE in human has been estimated as 5.6 cases per 100,000 inhabitants [15]. In such an endemic setting as Basrah, the prevalence of *E. granulosus* in stray dogs was recorded at 14.7%, while the prevalence of hydatid cysts in slaughtered sheep was reported as 22% [16]. The insight from this research could be used to develop health promotion tools and to improve the delivery of a better health education strategy relevant to CE control in Iraq.

## Figures and Tables

**Table 1:** Socio-demographic characteristics of livestock farmers (N = 314) participated in cystic echinococcosis (CE) knowledge, awareness and practices survey in Basrah, Iraq.

Variables	Category	*n*	%
County	Abu Al-Kasib Al-Midaina Al-Qurnah Al-Zubair Shat Al-Arab Al-Basrah	64 47 16 44 112 31	20.4 15 5.1 14 35.7 9.8
Age (years)	<20 21–30 31–40 41–50 51–60 61–70 >71	16 36 77 91 67 32 5	1.9 11.5 24.5 29 21.3 10.2 1.6
Gender	Male Female	311 3	99.1 0.9
Residence	Urban Rural	63 251	20.1 79.9
Education level	Illiterate (cannot read and write) Primary Secondary University	78 158 67 11	24.9 50.3 21.3 3.5
Principle occupation	Butcher Civil servant Farmer Policeman Solider Teacher Retired	3 32 245 19 2 7 6	0.9 10.2 78.1 6.1 0.6 2.2 1.9
Livestock ownership *	Cattle Cattle & sheep Cattle & buffalo Sheep Buffalo Buffalo, cattle & sheep Other mixed livestock farms	87 56 55 35 33 14 34	27.7 17.8 17.5 11.2 10.5 4.5 10.8

* Multiple answers allowed.

**Table 2:** Descriptive results of livestock farmers’ practices relevant to CE prevention and control.

Theme	Variables	Category	*n*	%
Dog ownership (N = 314) *	Do you own dog on your farm?	Yes No	86 228	27.4 2.6
Dog management practices (N = 86) **	Do you tie up your dog?	Yes No	20 66	23.3 76.7
	How long you keep your dog tied?	In the day time Only for the night	17 3	85 5
	Do you let your dog access your house?	Never Rarely Sometime Often Always	48 7 15 7 9	55.8 8.2 17.4 8.1 10.5
	Do you feed your dog uncooked animal viscera?	Yes No	37 49	43 57
	How do you handle droppings/stools of your dog?	Leave it where they are Bury it Throw in water canal Throw in agriculture field	82 1 1 2	95.3 1.2 1.2 2.3
	How often do you wash hands after handling or feeding your dog?	Never Rarely Sometime Often Always	16 0 12 15 43	18.6 0 14 17.4 50
	Do you regularly de-worm your dog?	Yes No	3 83	3.5 96.5
	How often does your dog have contact with farm livestock?	Never Rarely Sometime Often Always	21 2 11 19 33	24.4 2.3 12.8 22 38.4
	How often do you or your family members play with your dog?	Never Rarely Sometime Often Always	41 1 18 19 7	47.7 1.2 20.9 22.1 8.1
Food and water sanitary handling practices (N = 314) *	How frequently do you wash hands before eating?	All the time Most of the time Some of the time Do not wash hands	213 91 6 4	67.8 29 1.9 1.3
	How do you eat food in your home?	By hands only With cutlery only By both hand and cutlery	96 57 161	30.6 18.1 51.3
	How do you clean raw vegetables before eating them?	Do not wash (peel outer leaves) Rinse using tap water Soak in water in the sink With detergent With river water With well water With RO ^¥^ treated water	31 144 86 42 4 2 5	9.8 45.9 27.4 13.4 1.3 0.6 1.6
	What is the source of water for your domestic use?	River Tap water Well RO^¥^ treated water	100 191 5 18	32 61 1.3 5.7
	Do you boil water for domestic use before drinking it?	Yes No	18 296	5.7 94.3

* % was calculated among all interviewed farmers (N = 314). ** % was calculated among farmers indicated that they own dogs on their farms (N = 86). ^¥^ RO: reverse osmosis.

**Table 3:** Descriptive results of livestock farmers’ (N = 314) knowledge about and awareness of sources of infection with CE.

Theme	Variables	Category	*n*	%
Knowledge about CE	Do you know that some disease could be transmitted from livestock to human?	Yes No	186 128	59.2 40.8
	Did you hear about echinococcosis disease?	Yes No	222 92	70.7 29.3
	Do you know if echinococcosis disease can be dangerous to human health?	Yes No	184 130	58.6 41.4
	Have you ever seen hydatid cysts like these pictures in the organs of animals?	Yes No Not sure	117 190 7	37.3 60.5 2.2
Awareness of CE sources of infection	Do you know how humans are infected by hydatid disease?	Does not know Contaminated food Contaminated hands Contaminated water Eating raw food Contact with dogs faeces	161 50 61 20 9 13	51.3 15.9 19.4 6.4 2.9 4.1
	Are you aware that buffalo, cattle, sheep and goats can be infected with hydatid disease?	Yes No Not sure	100 202 12	31.8 64.4 3.8
	Are you aware that it could be dangerous to eat raw vegetables contaminated with dog faeces?	Yes No	225 89	71.7 28.3
	To what level do you consider de-worming of your dogs as a priority for your dog’s health?	Essential High priority Low priority Medium priority Not a priority	11 10 29 6 30	12.8 11.6 33.7 7 34.9
	To what level do you consider de-worming of your dogs as a priority for your family health?	Essential High priority Low priority Medium priority Not a priority	13 11 29 8 25	15.1 12.8 33.7 9.3 29.1

## References

[1] Harandi M.F., Budke C.M., Rostami S. (2012). The monetary burden of cystic echinococcosis in Iran. PLoS Negl. Trop. Dis..

[2] Deplazes P., Rinaldi L., Alvarez Rojas C.A., Torgerson P.R., Harandi M.F., Romig T., Antolova D., Schurer J.M., Lahmar S., Cringoli G. (2017). Global distribution of alveolar and cystic echinococcosis. Adv. Parasitol..

[3] Torgerson P.R., Macpherson C.N. (2011). The socioeconomic burden of parasitic zoonoses: Global trends. Vet. Parasitol..

[4] Budke C.M., Deplazes P., Torgerson P.R. (2006). Global socioeconomic impact of cystic echinococcosis. Emerg. Infect. Dis..

[5] Tashani O.A., Zhang L.H., Boufana B., Jegi A., McManus D.P. (2002). Epidemiology and strain characteristics of *Echinococcus granulosus* in the Benghazi area of eastern Libya. Ann. Trop. Med. Parasitol..

[6] Saida L.A., Nouraddin A.S. (2011). Epidemiological study of cystic echinococcosis in man and slaughtered animals in Erbil province, Kurdistan Regional, Iraq. Tikrit J. Pure Sci..

[7] Kandeel A., Ahmed E.S., Helmy H., El-Setouhy M., Craig P.S., Ramzy R.M.R. (2004). A retrospective hospital study of human cystic echinococcosis in Egypt. East. Mediterr. Health J..

[8] Chahed M.K., Bellali H., Touinsi H., Cherif R., Ben Safta Z., Essoussi M., Kilani T. (2010). Distribution of surgical hydatidosis in Tunisia, results of 2001–2005 study and trends between 1977 and 2005. Arch. Inst. Pasteur Tunis.

[9] Macpherson C.N. (2005). Human behaviour and the epidemiology of parasitic zoonoses. Int. J. Parasitol..

[10] Sadjjadi S.M. (2006). Present situation of echinococcosis in the Middle East and Arabic North Africa. Parasitol. Inter..

[11] Faraj R.A., Muhsin S.S. (2013). Epidemiological study of surgical cases between 2000–2012 at the Hospital of Gastrointestinal and Liver diseases in Baghdad city. IOSR J. Dent. Med. Sci..

[12] Abdul Ameer F., Al-Hassani A., Benyan A.Z. (2013). Pulmonary hydatid cysts disease in south of Iraq: Short term outcome after surgical intervention. J. Postgrad. Med. Inst..

[13] Thweni M.M., Yassen L.J. (2015). Hepatic hydatidosis in man and livestock in Nassiriyah, Iraq. Intern. J. PharmTech Res..

[14] Sawady N.J., Al-Faddagh Z. (2012). Study of bile leak after hepatic hydatid cyst surgery in Basrah. Basrah J. Surg..

[15] Thamir M.S., Abood Q.A., Halboosh A.K. (2015). An epidemiology study on human cystic echinococcosis (hydatid disease) in Basrah province during 2000–2005. J. Int. Acad. Res. Multidiscip..

[16] Maktoof A.R., Abu Tabeekh M.A.S. (2015). Classification of Endemicity of Cystic Echinococcosis in Basra Governorate, Iraq. Savant J. Agric. Res..

[17] Buishi I., Walters T., Guildea Z., Craig P., Palmer S. (2005). Reemergence of canine *Echinococcus granulosus* infection, Wales. Emerg. Infect. Dis..

[18] Ibrahim M.M. (2010). Study of cystic echinococcosis in slaughtered animals in Al Baha region, Saudi Arabia: Interaction between some biotic and abiotic factors. Acta Trop..

[19] Romig T., Omer R.A., Zeyhle E., Hüttner M., Dinkel A., Siefert L., Elmahdi I.E., Magambo J., Ocaido M., Menezes C.N. (2011). Echinococcosis in sub-Saharan Africa: Emerging complexity. Vet. Parasitol..

[20] Seimenis A. (2003). Overview of the epidemiological situation on echinococcosis in the Mediterranean region. Acta Trop..

[21] Craig P.S., Larrieu E. (2006). Control of cystic echinococcosis/hydatidosis: 1863–2002. Adv. Parasitol..

[22] NGO Coordination Committee for Iraq (2015). Basrah Governorate Profile. http://www.ncciraq.org/images/infobygov/NCCI_Basra_Governorate_Profile.pdf.

[23] Possenti A., Manzano-Román R., Sánchez-Ovejero C., Boufana B., La Torre G., Siles-Lucas M., Casulli A. (2016). Potential risk factors associated with human cystic echinococcosis: Systematic review and meta-analysis. PLoS Negl. Trop. Dis..

[24] Buishia I.E., Njorogeb E.M., Bouamrac O., Craiga P.S. (2005). Canine echinococcosis in northwest Libya: Assessment of coproantigen ELISA, and a survey of infection with analysis of risk-factors. Vet. Parasitol..

[25] Varcasia A., Tanda B., Giobbe M., Solinas C., Pipia A.P., Malgor R., Carmona C., Garippa G., Scala A. (2011). Cystic echinococcosis in Sardinia: Farmers’ knowledge and dog infection in sheep farms. Vet. Parasitol..

[26] Li D., Gao Q., Liu J., Feng Y., Ning W., Dong Y., Tao L., Li J., Tian X., Gu J., Xin D. (2015). Knowledge, attitude, and practices (KAP) and risk factors analysis related to cystic echinococcosis among residents in Tibetan communities, Xiahe County, Gansu Province, China. Acta Trop..

[27] Acosta-Jamett G., Cleaveland S., Bronsvoort B.M., Cunningham A.A., Bradshaw H., Craig P.S. (2010). *Echinococcus granulosus* infection in domestic dogs in urban and rural areas of the Coquimbo region, north-central Chile. Vet. Parasitol..

[28] Mateus T.L., Niza-Ribeiro J., Castro A., Vieira-Pinto M. (2016). Limited knowledge about hydatidosis among farmers in northwest Portugal: A pressing need for a One Health approach. EcoHealth.

[29] Otero-Abad B., Torgerson P.R. (2013). Systematic review of the epidemiology of echinococcosis in domestic and wild animals. PLoS Negl. Trop. Dis..

[30] Asfaw A., Afera B. (2014). Prevalence of hydatid cyst in cattle at municipal abbatoir of Shire. J. Vet. Sci. Technol..

[31] Torgerson P.R. (2014). Helminth-Cestode: *Echinococcus granulosus* and *Echinococcus mutilocularis*. Encycl. Food Saf..

[32] Chaâbane-Banaoues R., Oudni-M’rad M., Cabaret J., M’rad S., Mezhoud H., Babba H. (2015). Infection of dogs with *Echinococcus granulosus*: Causes and consequences in an hyperendemic area. Parasit. Vectors.

[33] Harandi MF., Moazezi S.S., Saba M., Grimm F., Kamyabi H., Sheikhzadeh F., Sharifi I., Deplazes P. (2011). Sonographical and serological survey of human cystic echinococcosis and analysis of risk factors associated with seroconversion in rural communities of Kerman, Iran. Zoonoses Public Health.

[34] Yang Y.R., Sun T., Li Z., Zhang J., Teng J., Liu X., Liu R., Zhao R., Jones M.K., Wang Y. (2006). Community surveys and risk factor analysis of human alveolar and cystic echinococcosis in Ningxia Hui Autonomous Region, China. Bull. World Health Organ..

[35] Qaqish A.M., Nasrieh M.A., Al-Qaoud K.M., Craig P.S., Abdel-Hafez S.K. (2003). The seroprevalences of cystic echinococcosis, and the associated risk factors, in rural-agricultural, bedouin and semi-bedouin communities in Jordan. Ann. Trop. Med. Parasitol..

[36] El Berbri I., Ducrotoy M.J., Petavy A.F., Fassifihri O., Shaw A.P., Bouslikhane M., Boue F., Welburn S.C., Dakkak A. (2015). Knowledge, attitudes and practices with regard to the presence, transmission, impact, and control of cystic echinococcosis in Sidi Kacem Province, Morocco. Infect. Dis. Poverty.

